# The Role of Thermal Immunomodulation in Postoperative Wound Repair with a Focus on Hepatic Surgery

**DOI:** 10.3390/ijms27031473

**Published:** 2026-02-02

**Authors:** Barbara Pietrzyk, Jedrzej Mikolajczyk, Aleksander Joniec, Tomasz Fajferek, Seweryn Kaczara

**Affiliations:** Department of Medical Biophysics, Faculty of Medical Sciences in Katowice, Medical University of Silesia, 40-752 Katowice, Poland

**Keywords:** thermal immunomodulation, liver, heat shock proteins (HSP), postoperative inflammation, damage-associated molecular patterns (DAMPs), wound healing immunology

## Abstract

Controlled local hyperthermia supports postoperative wound healing in liver surgery by stimulating metabolism, angiogenesis, and immune responses through the induction of heat shock proteins (HSPs) and modulation of Damage-Associated Molecular Patterns (DAMPs). This study evaluates the impact of thermal modulation on immune processes during abdominal wound repair, specifically analyzing the role of HSPs and immune activation pathways. A narrative review of the literature from 2010 to 2025 was conducted to summarize molecular mechanisms regarding temperature, HSP activation, cytokine expression, and DAMPs, excluding studies conducted solely in animal models. The results indicate that precise local hyperthermia in postoperative abdominal wounds activates HSPs as well as inflammasome and Toll-like receptor (TLR) pathways, modulating immune and cytokine responses depending on the type and depth of tissue injury. Consequently, such thermoimmunomodulation stabilizes immune cell functions, optimizes the balance between inflammation and regeneration, and minimizes the risk of postoperative complications to support effective wound healing.

## 1. Introduction

Thermoimmunomodulation represents an innovative approach to enhancing surgical wound healing. Postoperative wound repair in the abdominal cavity is a complex, multi-stage process comprising hemostasis, inflammation, proliferation, and remodeling. Scientific evidence to date indicates that there is no simple, linear cause-and-effect relationship between local temperature control and clinical outcomes of postoperative wound healing. In recent years, a clear shift has been observed in wound healing research toward the analysis of temperature as an active regulator of the wound microenvironment, capable of influencing tissue perfusion, immune responses, and the dynamics of reparative processes [1]. Therefore, the elements discussed in this paper are presented as interrelated components of tissue responses to thermal stress, which together may promote the balance between inflammation and recovery and promote effective healing of postoperative wounds, with particular emphasis on wounds after liver surgery. The proper progression of these phases is essential for effective tissue regeneration and restoration of full functionality. Increasing attention has been directed toward the role of body temperature particularly the maintenance of normothermia in the perioperative period—as a critical factor influencing the efficiency of wound healing [2]. The biological response to a thermal stimulus is dose-dependent and includes the intensity, duration, and localization of temperature exposure. The profile of this response may differ significantly depending on the type and depth of tissue injury as well as the local wound microenvironment, including the degree of perfusion and immune cell activity. The temperature of a healing postoperative wound may be considered an important indicator of the external wound microenvironment, as supported by studies showing that monitoring temperature curves using smart dressings can serve both as a predictive factor for sepsis and as a tool for assessing treatment outcomes [3].

Thermoregulatory disturbances occurring during and after abdominal surgery adversely affect the immune response and may lead to postoperative complications. Maintaining normothermia (approximately 37 °C) is now a standard in surgical practice, consistent with Enhanced Recovery After Surgery (ERAS) protocols, significantly reducing the risk of infection and shortening the length of hospitalization [4].

For example, intensive systemic warming in patients undergoing elective laparotomy has been associated with a more favorable immunologic profile, fewer complications, and faster recovery compared with patients in whom active warming was not applied [5]. Therefore, it can be concluded that even mild hypothermia may adversely affect hemostasis and thereby disrupt the wound healing process [2,6]. A postoperative wound that fails to heal within 4–6 weeks is classified as a chronic wound. This condition is often associated with persistent inflammation, an imbalance between tissue degradation and regeneration, and frequently the presence of a bacterial biofilm, which sustains the inflammatory process and hinders progression to subsequent phases of healing [7]. Local wound temperature—optimally around 37 °C, is critical for cellular metabolic activity and effective immune responses. During the inflammatory phase, a temperature increase supports vasodilation and enhances the migration of immune cells to the site of injury [2,8]. Heat shock proteins (HSPs) play a pivotal role in this process by stabilizing cellular proteins, protecting against damage, and supporting repair mechanisms. Their expression increases under thermal stress, promoting the proliferation and migration of keratinocytes, fibroblasts, and endothelial cells, thereby facilitating faster re-epithelialization and angiogenesis [9]. Heat shock proteins regulate inflammatory responses by negatively modulating the production of pro-inflammatory cytokines, including TNF-α, IL-6, and IL-1β, through transcriptional control mechanisms and regulation of inflammasome activity, thereby contributing to the attenuation of inflammatory signaling [10,11]. In addition, HSPs regulate the activity of matrix metalloproteinases and growth factors (e.g., VEGF), thereby supporting extracellular matrix (ECM) remodeling and neovascularization [12]. Thus, maintaining optimal wound temperature, or its moderate elevation, may accelerate healing by stimulating the activity of reparative cells and promoting angiogenesis [2].

The aim of this study was to analyze the effects of thermal modulation on immune processes and postoperative wound healing in the abdominal cavity, with a particular focus on the role of heat shock proteins and immune activation pathways in tissue repair. In contrast to previously published reviews that focus separately on general wound-healing mechanisms, perioperative thermoregulation strategies, or the molecular biology of heat shock proteins, the present review integrates these domains into a unified, mechanistic framework. Existing literature has predominantly addressed either clinical aspects of maintaining normothermia during surgery or the isolated cellular functions of heat shock proteins under stress conditions [13,14,15]. The current review extends beyond these approaches by linking local and systemic temperature modulation in abdominal surgical wounds with immune-regulatory and molecular signaling pathways, including Toll-like receptor activation, NLRP3 inflammasome signaling, and cytokine-driven transitions between inflammatory and regenerative phases. By combining immunological, molecular, and clinical perspectives, this work not only summarizes the current state of knowledge but also identifies relevant translational gaps and highlights the therapeutic potential of thermoimmunomodulation, including temperature-guided wound monitoring and targeted modulation of HSF1/HSP pathways to optimize postoperative wound healing.

## 2. Results

### 2.1. Local Wound Temperature and the Healing Process

In the context of liver surgery, optimal local wound temperature is a key factor modulating the healing process. During the first postoperative day, a physiological, moderate increase in local wound temperature of an acute nature is observed, reflecting an inflammation-driven response characterized by increased immune cell metabolism, prostaglandin-mediated vasodilation, and transient recruitment of neutrophils and macrophages, which is self-limiting and gradually resolves as perfusion stabilizes and tissue metabolism normalizes during the transition into the proliferative and remodeling phases [16]. A wound that fails to heal within approximately five weeks should be considered chronic, reflecting a persistent inflammatory state leading to progressive tissue degradation and disruption of the homeostasis between extracellular matrix (ECM) breakdown and reconstruction. Cutaneous wound healing proceeds through four main phases: hemostasis, inflammation, proliferation, and remodeling—each requiring precise integration of cellular and molecular processes [17]. A disruption of the skin barrier that is clinically assessed as painless and without signs of inflammation may still harbor underlying pathologies, such as bacterial biofilm, chronic inflammation, subclinical infection, or ischemia, which are not detectable in standard examination. In chronic wounds, biofilm exacerbates the inflammatory response through cytokine and protease activation, thereby impeding progression to the proliferative and remodeling phases [18]. In this context, chronic wounds are characterized by excessive matrix metalloproteinase (MMP) activity, reduced levels of growth factors such as vascular endothelial growth factor (VEGF) and epidermal growth factor (EGF), and persistently elevated pro-inflammatory cytokines, including TNF-α, IL-1β, and IL-6. This dysregulation of the wound microenvironment leads to an “inflammatory trap”, in which healing is arrested at the inflammatory phase, preventing progression to proliferation and remodeling due to prolonged exposure to disrupting factors such as biofilm, necrosis, and hypoxia [19]. Local temperature of a surgical wound is a critical determinant of effective skin healing. Maintaining an optimal temperature (around 37 °C) supports the metabolism of reparative cells, regulates the inflammatory response, and promotes proliferation.

Heat shock proteins (HSPs) play a central cytoprotective role by stabilizing cellular structures and participating in repair processes, thereby linking thermoregulatory mechanisms with the molecular control of tissue regeneration [20]. Local wound temperature rises during the inflammatory phase due to vasodilation and increased blood perfusion, leading to characteristic tissue warmth and erythema, and enhancing the transport of oxygen and immune cells to the injured area [21]. During this phase, phagocytic cells, particularly macrophages and neutrophils, are activated by pathogen-associated molecular patterns (PAMPs) and secrete pro-inflammatory cytokines—TNF-α (Tumor Necrosis Factor alpha), IL-1β (Interleukin 1 beta), and IL-6 (Interleukin 6)—which amplify inflammation and recruit additional immune cells to the wound [22]. In chronic wounds, the presence of bacterial biofilm sustains activation of pro-inflammatory M1 macrophages and prolongs cytokine production, thereby maintaining inflammation and elevating local tissue temperature [23]. Temperature influences the physicochemical properties of cell membranes, vascular permeability, and the diffusion rate of oxygen and nutrients within the wound [24]. Maintaining local wound temperature near 37 °C optimizes the activity of enzymes involved in collagen synthesis, fibroblast migration, and angiogenesis [25]. Local skin tissue temperature changes dynamically throughout the wound healing process. Local hypothermia (below 33–35 °C) can inhibit cell proliferation and prolong healing time, whereas moderate temperature elevation (36.5–38.5 °C) enhances metabolic activity, promotes angiogenesis, and modulates the inflammatory response. Concurrently, thermal stress induces the expression of heat shock proteins (HSPs), which play protective and regulatory roles in cellular and immune responses [26].

### 2.2. Wound Microenvironment/Immune Regulation

After skin injury, perfusion is disrupted, leading to heat loss and local hypothermia. Maintaining physiological temperature restores metabolic homeostasis and supports the function of reparative cells. Studies have shown that local wound heating enhances macrophage activity, keratinocyte proliferation, and collagen production [27]. Postoperative wound healing is a complex process comprising several stages, including the inflammatory response, cellular proliferation, and tissue remodeling. During the inflammatory phase, the transforming growth factor-beta/Smad (TGF-β/Smad) signaling pathway plays a pivotal role, as it establishes the conditions necessary for regeneration by regulating the inflammatory response and controlling extracellular matrix production [28]. Subsequently, the signaling pathway of Rho proteins, members of the small GTPase family (Rho GTPases), is activated. This pathway governs cytoskeletal reorganization, thereby facilitating the migration of fibroblasts and keratinocytes to the site of injury [29]. During the proliferative phase, the phosphatidylinositol 3-kinase/protein kinase B (PI3K/Akt) pathway plays a critical role by promoting cell survival and proliferation, thereby accelerating tissue reconstruction and wound healing [30]. In parallel, the Wnt/β-catenin signaling pathway is activated. By inducing the expression of genes responsible for keratinocyte and fibroblast proliferation and migration, this pathway stimulates granulation tissue formation and further accelerates tissue regeneration [31]. The coordinated activity of these signaling pathways ensures effective skin regeneration following surgical intervention. The process of re-epithelialization plays a critical role during the tissue regeneration phase following skin injury. It involves the migration, proliferation, and differentiation of epithelial cells, which collectively enable wound closure and restoration of epidermal barrier integrity and function. Effective re-epithelialization is essential for proper postoperative wound healing and for preventing infection and further complications [28].

By contrast, in chronic wounds, persistently elevated levels of proinflammatory cytokines—such as interleukin-1β (IL-1β), interleukin-6 (IL-6), and tumor necrosis factor-alpha (TNF-α)—impede the transition of the wound into the proliferative phase [32]. This leads to a disruption of immunological homeostasis between pro- and anti-inflammatory cytokines. Proinflammatory cytokines, such as tumor necrosis factor-alpha (TNF-α), interleukin-1 beta (IL-1β), and interleukin-6 (IL-6), predominate, maintaining immune cells—primarily macrophages and neutrophils in a state of persistent activation [33]. Macrophages fail to effectively transition from the proinflammatory (M1) to the reparative (M2) phenotype, which in turn suppresses the synthesis of transforming growth factor-beta (TGF-β) and vascular endothelial growth factor (VEGF)—key mediators of proliferation and angiogenesis [34].

Neutrophils play an important role by forming neutrophil extracellular traps (NETs), composed of their own DNA and enzymes, which physiologically function to neutralize pathogens. However, when produced in excess, NETs may also exert detrimental effects. Excessive or uncontrolled NET formation during chronic inflammation leads to the release of large amounts of reactive oxygen species (ROS)—including hydrogen peroxide (H_2_O_2_), superoxide anion (O_2_^−^•), and hydroxyl radical (•OH) as well as proteolytic enzymes, such as matrix metalloproteinases (MMPs). Collectively, these factors exacerbate tissue injury, sustain inflammation, and promote the progression of pathological alterations within the affected tissues [35]. Tissue perfusion disturbances lead to local hypoxia, which sustains activation of the hypoxia-inducible factor 1-alpha (HIF-1α) pathway, promoting persistent inflammation and colonization of the wound by bacteria capable of forming biofilms [36]. Bacterial biofilms, complex structures of microorganisms embedded in an extracellular polysaccharide matrix, provide protection against immune system mechanisms and antibiotic therapy. Consequently, prolonged and excessive biofilm persistence contributes to chronic inflammation, thereby impairing proper wound healing [37]. Chronic inflammation and the presence of biofilms may occur without the typical clinical signs of infection; however, they are often accompanied by elevated wound temperature, which complicates diagnosis, promotes treatment resistance, and contributes to prolonged impairment of healing. Although visible symptoms may be absent, histological analysis typically reveals inflammatory infiltration, microcirculatory disturbances, and a deficit of regenerative cells [38]. It has been demonstrated that bacterial biofilms, present in the majority of chronic wounds, inhibit tissue regeneration and retain the capacity to rapidly reestablish themselves even after surgical debridement [39].

The absence of significant changes in body temperature in isolated cases does not rule out the presence of biofilm or a hidden infection; therefore, the interpretation of such findings should always be considered in the context of other clinical and diagnostic parameters [40].

Understanding the immunological underpinnings of chronic wounds enables the implementation of targeted immunomodulatory therapies. The presence of biofilm and persistent inflammation is currently recognized as a major factor delaying wound healing, which is why the assessment of inflammatory biomarkers and wound microbiome profiling is increasingly recommended prior to selecting appropriate therapy [41].

### 2.3. Temperature and Modulation of the Immune Response

Local tissue temperature represents a critical parameter regulating the immune response within the wound, directly influencing immune cell activity as well as the dynamics of inflammatory and regenerative processes [42]. Maintaining a moderate increase in temperature (approximately 37–39 °C) promotes the transition of macrophages from the proinflammatory M1 phenotype to the reparative M2 phenotype, leading to increased secretion of anti-inflammatory cytokines, such as interleukin-10 (IL-10), and enhanced production of transforming growth factor—beta (TGF-β)—a key mediator of the proliferative and remodeling phases [43,44].

Heat induces the expression of heat shock proteins, particularly HSP70 (Heat Shock Protein 70) and HSP90 (Heat Shock Protein 90), which function as molecular chaperones stabilizing proteins during cellular stress. Simultaneously, HSPs act as damage-associated molecular patterns (DAMPs), binding to Toll-like receptors (TLR2 and TLR4) on the surface of immune cells and activating the NF-κB (nuclear factor kappa-light-chain-enhancer of activated B cells) transcriptional pathway, which regulates the expression of downstream cytokines [45]. Activation of this pathway under conditions of moderate thermal stress exhibits a regulatory effect, promoting a balance between immunity and regeneration and limiting chronic inflammatory activation. It also serves as an important protective mechanism against persistent tissue damage [46]. Thus, elevated temperature modulates macrophage activation, the production of pro- and anti-inflammatory cytokines, and oxidative stress. Moderate temperature elevation (local hyperthermia) may accelerate the transition of the wound from the inflammatory to the proliferative phase, thereby supporting tissue regeneration [47]. Conversely, local hypothermia in ischemic and edematous wounds inhibits the migration of neutrophils and macrophages, impairs phagocytosis, and reduces the activity of fibroblasts and keratinocytes, thereby preventing the transition of the wound into the proliferative phase and perpetuating inflammation [48,49]. Thus, it can be concluded that temperature is not merely a passive indicator of the inflammatory process but an active factor modulating the immune response, influencing healing efficiency both through physiological signaling pathways and via direct effects on intracellular molecular processes- Table 1.

Understanding the functions of these cellular components enables a deeper insight into the mechanisms of wound repair and the development of targeted therapies for the treatment of chronic and hard-to-heal postoperative wounds. Table 1 summarizes the key growth factors, cytokines, heat shock proteins, and extracellular matrix components that directly influence the regulation of the immune response, thereby contributing to the healing of mechanically damaged cellular layers. A body temperature of 38 °C induces mild thermal stress, activating heat shock proteins (HSP70 and HSP90), which, as molecular chaperones, stabilize and repair denatured proteins through refolding after damage. This prevents protein aggregation and allows their return to an active conformation. This represents a physiological, adaptive protective response of cells, without pathological features [50]. In contrast, at temperatures of 39–40 °C, moderate fever occurs, accompanied by increased production of proinflammatory cytokines (IL-1β, TNF-α), which further stimulate the immune response and enhance cellular metabolism and tissue perfusion. Although heat shock proteins continue to provide protective functions, prolonged exposure to elevated temperatures impairs the function of cells particularly sensitive to oxidative stress, such as keratinocytes, fibroblasts, T lymphocytes, and M2 macrophages. This leads to significant disruption of regenerative processes and prolongation of the inflammatory state [51]. At temperatures exceeding 40 °C, a pathological state develops in which the heat shock protein response is minimal, typically resulting in protein denaturation and disruption of enzymatic functions. The organism activates thermoregulatory mechanisms; however, in extreme cases, apoptosis or necrosis of cells occurs as a means to reduce temperature [52]—Table 2.

**Figure 1:**
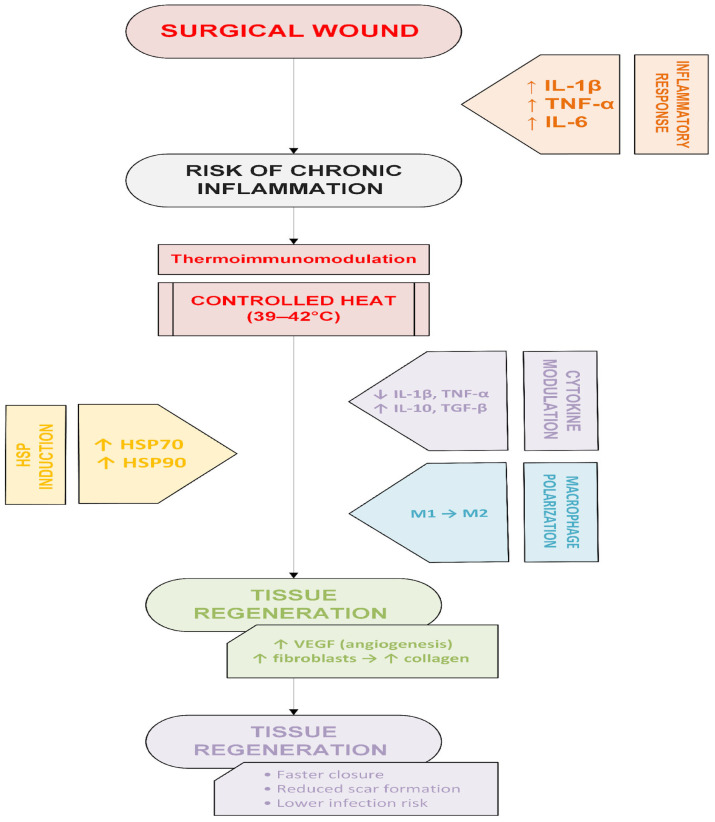
The wound inflammatory response and the role of cytokines and macrophages in wound healing [56].

Heat Shock Factor 1 (HSF1) is a key transcription factor activated in response to cellular stress, such as elevated temperature, oxidative stress, or hypoxia. Upon trimerization and phosphorylation, HSF1 translocates to the nucleus, where it binds to specific heat shock elements (HSE) in the promoters of heat shock protein (HSP) genes, activating their transcription, particularly HSP70 and HSP90, which perform protective functions [53]. Induction of HSP expression by HSF1 safeguards cellular proteins from denaturation and damage caused by oxidative stress [54]. At the immunological level, HSP70 functions as a damage-associated molecular pattern (DAMP) signal, activating Toll-like receptors TLR2 and TLR4 on innate immune cells such as macrophages and neutrophils. This leads to controlled activation of the NF-κB (nuclear factor kappa-light-chain-enhancer of activated B cells) pathway and the secretion of cytokines, including TNF-α and IL-6 [55]. Notably, moderate temperature elevation favorably modulates the wound environment by improving perfusion and activating M2 macrophages, thereby accelerating the proliferative phase of wound healing [56]—Figure 1.

### 2.4. Molecular Cellular Response to Thermal Stress

HSP70 is induced in wound cells and modulates the immune response by inhibiting excessive production of proinflammatory cytokines, such as TNF-α and IL-1β, thereby contributing to the limitation of inflammation [57]. NF-κB serves as a key regulator of inducible gene expression in the immune system. In response to thermal stress, signaling pathways such as NF-κB (Nuclear Factor kappa-light-chain-enhancer of activated B cells) are also activated, regulating the expression of genes involved in inflammation and tissue regeneration [58]. Furthermore, thermal stress induces the production of growth factors, including vascular endothelial growth factor (VEGF), which support angiogenesis and reperfusion of damaged tissue within the wound [59]. Heat shock proteins also influence cytoskeletal stabilization and enhance keratinocyte migration, which is crucial for wound closure and epidermal reconstruction [60]. Ultimately, modulation of the thermal stress response in postoperative wounds may serve as a therapeutic target, enabling optimization of healing processes and reduction in inflammatory and infectious complications [61].

Thermal stress (temperature ≥ 37.5–39 °C) activates transcription factors from the Heat Shock Factor (HSF) family, particularly HSF1. HSF1 trimerizes, undergoes phosphorylation, and binds to heat shock elements (HSE) in the promoters of HSP genes [62]. Emerging therapeutic strategies include the use of preparations containing growth factors (e.g., platelet-derived growth factor, PDGF) and stem cell-derived exosomes, which support the transition to the proliferative phase and promote angiogenesis [63]. In abdominal wounds, it has been demonstrated that modulation of the immune response by HSP70 and other HSPs supports angiogenesis and extracellular matrix stabilization, which are essential for effective wound closure and the prevention of complications such as leaks and infections. Accordingly, therapies targeting the modulation of HSP expression and the optimization of local wound temperature may significantly improve healing outcomes following abdominal procedures, particularly in difficult-to-treat postoperative wounds [64].

In chronic wounds, excessive activity of matrix metalloproteinases (MMPs) leads to the degradation of newly formed extracellular matrix and growth factors, resulting in tissue destruction and impaired healing. Through the evaluation of novel therapeutic strategies—including MMP inhibitors, M1/M2 macrophage modulators, and targeted anti-biofilm therapies—it has recently become possible to gain a better understanding of the molecular pathways underlying chronic inflammation [65]. For example, inhibition (or modulation) of small heat shock proteins, particularly HSP27, to disrupt their interactions with actin (thereby limiting cytoskeletal reorganization and cell migration) as well as their influence on the expression of matrix metalloproteinases (MMPs), may help reduce pathological tissue remodeling [66].

### 2.5. How Heat Shock Proteins and Temperature Modulate Postoperative Wound Healing

Postoperative wounds following abdominal surgery, particularly transverse incisions in the liver region, are characterized by a high risk of hypoxia and a complex inflammatory response resulting from tissue contact with bodily fluids and potential bacterial contamination. In these wounds, thermal stress and the induction of heat shock proteins (HSPs) play a critical role in protecting hepatocytes and mesothelial cells from oxidative stress and cytotoxic damage [67].

Heat shock proteins (HSPs), particularly HSP70 and HSP90, are key molecular chaperones that stabilize proteins under stress conditions, prevent their aggregation, and facilitate proper folding. In the context of surgical wounds, they are strongly activated by local thermal stress as well as by hypoxia and inflammation [68]. HSP70 can function as an immunological danger signal (DAMP—damage-associated molecular pattern), activating dendritic cells, macrophages, and neutrophils via Toll-like receptors TLR2 and TLR4, leading to controlled proinflammatory cytokine activation and the initiation of a localized immune response [69]. Simultaneously, HSPs can suppress excessive immune system activation by limiting NF-κB (nuclear factor kappa-light-chain-enhancer of activated B cells) activity, reducing the expression of TNF-α and IL-6, thereby preventing prolonged inflammation and supporting the transition of the wound into the proliferative phase [70]. In wound healing models, exogenous stimulation of HSP70 expression has been shown to enhance angiogenesis, increase the presence of M2 macrophages, and accelerate skin re-epithelialization. The most relevant HSPs in the context of wound healing are HSP70, HSP90, and small HSPs, such as HSP27 [71]. The most important effects of HSP expression in wound cells include cytoskeletal and structural protein stabilization (HSP27, HSP70), inhibition of the apoptotic cascade (HSP70 suppresses activation of caspase-3 and caspase-9), modulation of cytokine expression (HSPs influence IL-6, TNF-α, TGF-β), and the induction of immunological and regenerative tolerance [72]—Table 3.

Optimal local wound temperature (approximately 37 °C) and the role of heat shock proteins (HSPs) form a synergistic system supporting the wound healing process. Temperature regulates metabolism and inflammatory response, while HSPs protect cells from damage and modulate immunological and reparative cellular functions. In the context of the wound, regulation of HSP interactions with surface receptors—such as TLR4 and CD91—is very important, leading to activation of MAPK (Mitogen-Activated Protein Kinase) and NF-κB (Nuclear Factor kappa-light-chain-enhancer of activated B cells) signaling pathways. The most potent immunological mechanism activating HSP expression in the wound is signaling via Toll-like receptors (TLRs), especially TLR2 and TLR4 [73]. Under conditions of tissue damage and the presence of pathogens or biofilm, immune cells (macrophages and dendritic cells) specifically recognize danger signals—DAMPs (damage-associated molecular patterns). One such DAMP can be HSP70 itself, which, upon release into the extracellular space, acts as a ligand for TLR2 and TLR4. Activation of these receptors triggers an NF-κB–dependent cascade, leading to the expression of genes encoding proinflammatory cytokines (TNF-α, IL-1β, IL-6) while also stimulating the cells to express their own HSPs as a protective mechanism [74]. Activation of these receptors triggers an NF-κB (nuclear factor kappa-light-chain-enhancer of activated B cells) dependent cascade, leading to the expression of genes encoding proinflammatory cytokines (TNF-α, IL-1β, IL-6) while also stimulating cells to express their own HSPs as a protective mechanism [75]. Additionally, hypoxia within the wound activates the transcription factor HIF-1α (hypoxia-inducible factor 1-alpha), which can indirectly influence HSP pathway activation by enhancing oxidative stress and stabilizing damaged proteins [76]. Activation of host defense peptides (HDPs), also known as antimicrobial peptides (AMPs), has also been observed. These peptides function bidirectionally as both antibacterial agents and immunomodulators, participating in the modulation of signaling pathways involved in pathological as well as physiological processes, such as wound healing. HDPs can selectively regulate gene expression and modify the functions of epithelial and immune cells, thereby influencing the shaping of the immune response [77]. In response to elevated temperature and cellular stress, cells within the wound synthesize HSP70 and HSP90, while also stimulating the production of vascular endothelial growth factor (VEGF) and platelet-derived growth factor (PDGF), thereby supporting angiogenesis, extracellular matrix remodeling, and cellular protection against apoptosis [10].

HSP70 accelerates fibroblast migration and the synthesis of type I and III collagen, HSP27 promotes keratinocyte proliferation and their re-epithelialization capacity, while HSP90 stabilizes VEGF/VEGFR complexes in endothelial cells, supporting angiogenesis [78]. Maintaining an optimal local temperature is essential for enzymatic and cellular activity and activates the heat shock response, thereby stimulating the expression of heat shock proteins. In turn, HSPs stabilize biochemical processes within the wound, protect cells from stress, and modulate immune responses and tissue regeneration [15].

### 2.6. Immunophysiology of Postoperative Liver Wound Healing in Response to Surgical Injury

Fever following liver surgery is common, typically lasting 1–3 days, but it may persist longer, particularly in cases of abscess formation, infection, or hepatic parenchymal necrosis [79]. The mechanism of fever is complex and reflects both the systemic response to surgical trauma and the activation of innate immune pathways triggered by tissue injury. Surgical stress leads to the release of damage-associated molecular patterns (DAMPs), including adenosine triphosphate (ATP) and high mobility group box 1 (HMGB1) [80], which are recognized by pattern recognition receptors and initiate inflammatory signaling cascades. These DAMP-driven pathways contribute to cytokine release and downstream thermoregulatory responses, linking local tissue damage with systemic immune activation in the perioperative setting [81]. Additionally, the presence of hematomas or necrosis may sustain or exacerbate inflammation through inflammasome activation and the secretion of pyrogenic cytokines (e.g., IL-1β, IL-6, TNF-α) [82]. In patients with compromised immunity, such as those undergoing chemotherapy or with liver cirrhosis, the febrile response may be attenuated or delayed, even in the presence of inflammation. Therefore, in this population, it is crucial not only to measure body temperature but also to monitor inflammatory parameters, such as C-reactive protein (CRP), IL-6, and procalcitonin (PCT), enabling differentiation between physiological postoperative fever and infection [83,84]—Table 4.

#### Gradual Immunological and Pyrogenic Response

Abdominal surgery, particularly procedures involving liver access, requires incision through multiple anatomical structures, whose injury triggers activation of specific immunological pathways. The deeper the layers affected—especially muscles, deep fascia, peritoneum, and liver parenchyma—the stronger the inflammatory response, resulting in more pronounced systemic manifestations, such as postoperative fever [85,86]—Table 5.

Tissue injury leads to the release of non-pathogen-derived molecules, known as DAMPs (Damage-Associated Molecular Patterns), such as ATP, HMGB1, mitochondrial DNA (mtDNA), and HSPs. These molecules are recognized by innate immune receptors—TLR4, TLR9, and TLR2—primarily expressed on phagocytes, dendritic cells, and endothelial cells. Activation of these receptors triggers intracellular signaling cascades and transcription of proinflammatory genes. The NLRP3 inflammasome plays a particularly important role in this process [87]—a cytoplasmic protein complex activated by danger signals (DAMPs) as well as mechanical, metabolic, and infectious stimuli [88]. Its activation in macrophages (e.g., Kupffer cells in the liver) converts pro-caspase-1 into active caspase-1, resulting in maturation of interleukin IL-1β and other proinflammatory cytokines, including IL-6, TNF-α, and IL-18. These mediators, upon reaching the central nervous system, induce expression of COX-2 in hypothalamic endothelial cells, leading to increased production of PGE_2_ and elevation of the body temperature set point, thereby triggering fever [89]. Vascular endothelial cells, in response to cytokines and the presence of DAMPs, also upregulate adhesion molecules (ICAM-1, VCAM-1), facilitating leukocyte recruitment to the site of injury. This response can remain localized but often becomes systemic (SIRS), particularly in the presence of parenchymal tissue necrosis (e.g., liver) or infection. Skeletal muscles and the liver are considered highly immunogenic structures because they contain abundant mitochondria, ATP, and heat shock proteins, making them potent sources of DAMPs [90]. Early postoperative fever (≤48 h, usually <38.5 °C) is most often a physiological response to surgical trauma and resolves spontaneously. Diagnostic evaluation is typically limited to clinical assessment and is extended only in cases of fever lasting >48–72 h, high-grade temperature, or signs of infection or sepsis. Management primarily involves observation and symptomatic treatment, with antibiotics reserved exclusively for confirmed infections [79]. Postoperative fever persisting above 38.5 °C for more than 48–72 h, often recurring after an initial decrease and accompanied by signs such as erythema, swelling, purulent wound discharge, severe pain, tachycardia, and elevated inflammatory markers, primarily C-reactive protein (CRP) and procalcitonin (PCT) may indicate pathological processes, including impaired wound healing or inflammatory complications that require urgent intervention. CRP has been shown to correlate with the response to antibiotics following abdominal surgeries, while PCT levels increase after major liver procedures [91].

### 2.7. Thermal Modulation of Wound Healing: Clinical Applications

A moderate increase in local wound temperature from the physiological body temperature of approximately 36.6 °C to 37–38 °C can significantly accelerate healing by activating reparative cells and stimulating angiogenesis [92]. In vivo studies have measured wound temperature using skin thermometers or thermal sensors placed directly on the wound surface, allowing precise monitoring and control of local temperature. Warming the wound to 38 °C for a defined period enhances microcirculation and supports regenerative processes, as evidenced by increased markers of cell proliferation and neovascularization [93]. A retrospective study involving 4000 patients undergoing liver resection demonstrated that moderate hypothermia, defined as a body temperature below 36 °C, was not significantly associated with an increased rate of surgical site infections (SSI)—7.0% in the hypothermic group versus 6.3% in the normothermic group (body temperature ≥ 36 °C). These findings suggest that active perioperative normothermia maintenance strategies can effectively mitigate the adverse effects of hypothermia on postoperative infection risk [94]. Conversely, a 1 °C decrease in body temperature increases blood loss by 16% and transfusion requirements by 22%. Recent studies confirm that hypothermia exacerbates bleeding and the need for transfusions, which may indirectly impair wound healing [95]. A study by Luo et al. in patients undergoing laparoscopic gastrectomy demonstrated that continuous active warming significantly reduced the incidence of intraoperative hypothermia (16%), decreased the occurrence of shivering (3% vs. 32%), accelerated extubation, and alleviated pain, indicating an overall improvement in postoperative recovery [96].

## 3. Discussion

In the context of surgical procedures, maintaining normothermia and actively managing patient temperature through continuous active warming significantly reduces the risk of intraoperative hypothermia, minimizes complications such as excessive blood loss, and accelerates recovery, thereby positively influencing the overall course of wound healing. Maintaining an optimal local wound temperature promotes cell proliferation, angiogenesis, and modulates inflammatory responses. Heat shock proteins play the main role in protecting cells from thermal stress and supporting regenerative processes. Chronic wounds are characterized by persistent inflammation and immune cell dysfunction, which impede healing. Local thermal regulation is therefore an important factor in accelerating tissue repair. Recent reviews focusing on thermal dynamics in wound healing emphasize that wound temperature should be regarded as a dynamic indicator of the local wound microenvironment rather than a static physical parameter. Changes in local temperature reflect alterations in tissue perfusion, metabolic activity, and immune cell function, thereby providing indirect information on the inflammatory and reparative status of the wound. This concept supports the view that thermal modulation influences wound healing through complex, context-dependent mechanisms rather than through a simple linear cause–effect relationship [97]. Studies indicate that chronic inflammation within a wound leads to disruptions in the tissue microenvironment, which is a key factor impeding wound healing. Furthermore, impaired microcirculation results in tissue hypoxia, promoting the persistence of inflammation and inhibiting reparative processes [98]. Additionally, a deficiency of progenitor stem cells in the wound vicinity limits cellular regeneration and tissue remodeling, as confirmed by recent studies on chronic wounds [99].

In recent years, there has been growing interest in the molecular and immunological mechanisms through which tissues respond to temperature changes in the context of wound healing, with temperature increasingly regarded as a dynamic component of the wound microenvironment rather than merely a physical parameter. From the perspective of postoperative wounds within the abdominal cavity, local thermal changes may reflect the balance between a physiological inflammatory response and early features of complications, including infection. In liver surgery, due to the high degree of tissue injury and the pronounced release of inflammatory mediators, the interpretation of temperature changes should consider the coexistence of regenerative and inflammatory processes, as reflected, among others, in the dynamics of markers such as interleukin-6. In parallel, advanced technologies enabling monitoring of wound parameters, including temperature, are being developed, which may support early assessment of the healing process and facilitate the identification of deviations from the normal postoperative course [2].

During hepatic surgical procedures, there is a sequential depth-dependent activation of the immune response. Injury to superficial layers such as the skin, subcutaneous tissue, and superficial fascia initiates a limited inflammatory response, primarily through local production of cytokines, including IL-6 and TNF-α. However, deeper tissue damage, particularly involving muscles, deep fascia, peritoneum, and liver parenchyma, results in massive release DAMPs, such as ATP, high-mobility group box 1 protein (HMGB1), mtDNA, and HSPs [100]. DAMP molecules are recognized by Toll-like receptors (TLRs), including TLR2, TLR4, and TLR9, expressed on the surface of monocytes, macrophages, and dendritic cells. Signaling through these receptors leads to activation of the NLRP3 inflammasome (NOD-Like Receptor Family Pyrin Domain Containing 3), a molecular complex responsible for the production of proinflammatory IL-1β, one of the key mediators of fever [101]. The NLRP3 inflammasome is a cytoplasmic protein complex activated by danger signals (DAMPs) as well as mechanical, metabolic, and infectious stimuli. Its activation leads to the conversion of pro-caspase-1 into active caspase-1, resulting in the maturation of IL-1β and IL-18 [102]. Released IL-1β, together with IL-6 and TNF-α, activates brain vascular endothelial cells, stimulating the expression of cyclooxygenase-2 (COX-2) and the synthesis of prostaglandin E_2_ (PGE_2_), which elevates the set point of body temperature in the hypothalamic thermoregulatory center [101]. Endothelial cells in the damaged tissue simultaneously exhibit increased expression of adhesion molecules, such as intercellular adhesion molecule 1 (ICAM-1) and vascular cell adhesion molecule 1 (VCAM-1), which facilitates leukocyte migration to the site of injury. This effect is important both for local control of the damage and for the development of a systemic inflammatory response (SIRS) [103].

A particularly intense pyrogenic response occurs in cases of liver and skeletal muscle injury. This is due to their high immunogenic potential, associated with the following: a high mitochondrial content (which increases the release of mtDNA and ATP as DAMP signals), the presence of numerous heat shock proteins (HSP60, HSP70), a high number of tissue macrophages (especially Kupffer cells), extensive vascularization and availability of endothelial cells (facilitating rapid propagation of inflammatory signals), and the ability to produce large amounts of cytokines in response to cellular stress and mechanical injury [104]. The development of HSF1 modulators is still an ambitious research area that has the potential to discover novel therapeutic approaches for pathological processes characterized by protein misfolding and aggregation, including improving the quality and time of postoperative wound healing [105]. As a result, surgical transection of these tissues triggers cytokine and pyrogenic cascades much more intensely than in other structures. This phenomenon explains the occurrence of early postoperative fever, often already within the first day after liver surgery, and it should be distinguished from an infection requiring antibiotic therapy. In clinical practice, monitoring the dynamics of IL-6, C-reactive protein (CRP), and procalcitonin (PCT) levels is helpful. Persistent or recurrent postoperative fever, especially above 38.5–39 °C, accompanied by tachycardia, elevated inflammatory markers, and signs of local infection, indicates a pathological course of wound healing, requiring prompt diagnostics and therapeutic intervention [106]. On the other hand, a moderate increase in the local wound temperature to the range of 37–38 °C supports healing processes by stimulating reparative cells and promoting angiogenesis, as confirmed by both experimental and clinical studies. In the context of surgical procedures, maintaining normothermia and actively managing the patient’s temperature through continuous active warming—significantly reduces the risk of intraoperative hypothermia, complications such as excessive blood loss, and accelerates recovery, positively influencing the overall course of wound healing. Clinical studies on local controlled wound heating indicate that moderate increases in wound temperature lead to enhanced local blood flow and an increase in lymphocyte activation within the wound area. These effects improve the delivery of oxygen and nutrients to damaged tissues and modulate the immune response, which may support reparative processes. Such findings confirm that local thermal modulation influences wound healing through effects on microcirculation and cellular responses rather than through a simple, direct cause-and-effect mechanism [107]. The effects of tissue warming, both local and systemic, indicate that controlled elevation of temperature may accelerate wound healing and reduce the risk of postoperative infections. These effects are primarily associated with improved microcirculation, increased tissue perfusion, and enhanced oxygen delivery, which together support a more effective immune response and activation of reparative processes within the wound. At the same time, there is a recognized need for further well-designed clinical studies to clearly define the optimal parameters and therapeutic range for the use of warming in clinical practice [108].

Current clinical evidence confirms that maintaining normothermia reduces the risk of immunological and infectious complications, although with properly managed warming, the impact of hypothermia may be minimal. Even mild cooling negatively affects hemostasis and can impair wound healing. Continuous Active Warming (CAW) strategies in abdominal surgeries have been shown to positively influence both the healing process and patient recovery [104,106]. There are no randomized clinical studies in humans directly assessing the impact of moderate hyperthermia (37–38 °C) on postoperative wound healing after liver surgery. However, maintaining perioperative normothermia and preventing hypothermia is well documented as beneficial for reducing complications such as the following: hemostatic disturbances, impaired immune response, surgical site infections, bleeding, and delayed healing [109].

The most important and potent immunological mechanism activating heat shock protein expression (HSP70 and HSP90) in wounds is the stimulation of TLR2 and TLR4 receptors on innate immune cells, which initiates the NF-κB signaling pathway and the inflammatory response. This activation is further supported by hypoxia via a HIF-1α–dependent mechanism [110]. Experimental studies have shown that local in vivo delivery of HSP70 significantly accelerates wound closure. This effect is primarily associated with enhanced macrophage-mediated phagocytosis, which facilitates more effective clearance of cellular detritus and promotes progression to the reparative phase. These findings support the concept that heat stress–induced HSP70 represents an important link between thermal stimuli and functional cellular responses within the wound microenvironment [111].

The optimal local wound temperature, together with the functions of heat shock proteins (HSPs), forms a complex, synergistic mechanism supporting the healing of skin tissues. While temperature regulates cellular metabolism and modulates the inflammatory response, HSPs play a main role in protecting cells from stress and damage, as well as in regulating immunological and regenerative repair processes.

Available evidence indicates that wound temperature measurement, particularly when combined with other local parameters, may serve as a useful prognostic indicator of postoperative wound healing. Dynamic changes in wound temperature reflect both the physiological stages of inflammatory and regenerative responses as well as the early features of pathological progression, including the development of infection. In this context, wound temperature is closely linked to local tissue perfusion, which determines the effectiveness of the immune response and oxygen delivery, thereby influencing the efficiency of reparative processes. These relationships highlight the relevance of temperature as an integrative parameter linking microcirculation, immunity, and wound healing, supporting further investigation into its application for monitoring and early assessment of postoperative wound healing.

This narrative review has several limitations. First, there are currently no randomized clinical trials directly evaluating controlled local hyperthermia in the context of postoperative wound healing following hepatic surgery, which limits causal inference. Second, the distinction between beneficial thermo-immunomodulatory effects and potentially harmful hyperthermic stress remains insufficiently defined, due to the lack of standardized temperature ranges and exposure parameters. Moreover, the cited literature is characterized by substantial heterogeneity in wound models, experimental settings, and outcome measures, which restricts direct comparison across studies.

Therefore, the presented findings should be interpreted as hypothesis-generating and highlight the need for well-designed clinical investigations.

The strength of evidence supporting temperature-dependent immunomodulation and heat shock protein-mediated wound repair remains heterogeneous and should be interpreted cautiously. Available clinical data in humans indicate a potential association between perioperative normothermia and reduced bleeding, fewer infections, and improved wound healing; however, direct evidence linking moderate local hyperthermia to improved postoperative wound healing after liver surgery is limited. Much of the mechanistic insight regarding DAMP signaling, inflammasome activation, and HSP induction derives from translational ex vivo human tissue studies and complementary animal or in vitro models. In accordance with the applied exclusion criteria, studies performed exclusively in animal models without demonstrated relevance to human molecular responses, as well as purely technical or non-mechanistic reports, were excluded from the analysis. Nevertheless, the scarcity of randomized human studies directly assessing temperature-driven modulation of wound biology highlights an important limitation of the current literature and underscores the need for future clinical trials integrating molecular endpoints with postoperative outcomes.

### Future Directions: Biomarker-Guided Thermal Therapies and Smart Technologies

To translate the molecular mechanisms of thermoimmunomodulation into clinical practice, future research must move beyond general normothermia maintenance toward precision medicine approaches. We propose a structured roadmap focused on biomarker-guided algorithms and the integration of smart surgical materials.

Proposal for Biomarker-Guided Thermal Dosing Current intraoperative warming protocols are largely empiric. We postulate that “thermal dosing” (duration and target temperature) should be personalized based on the patient’s inflammatory and regenerative status. Future clinical trials should evaluate the utility of the following candidate markers to guide thermal therapy:

- Systemic Inflammatory Markers (CRP, IL-6, Procalcitonin): Persistent elevation of PCT or IL-6 alongside fever (>38.5 °C) suggests an infectious etiology or pathological inflammation rather than physiological surgical stress [91,112]. In such cases, local hyperthermia is contraindicated. The goal is strict normothermia or targeted cooling to prevent excessive metabolic demand and DAMP release that could exacerbate the inflammatory cascade [106].
- Local Wound Microenvironment Markers (HSP70, VEGF): Chronic or hard-to-heal wounds often exhibit insufficient HSP induction and low VEGF levels [113,114]. Controlled local hyperthermia (37.5–38.5 °C) applied for defined intervals could be used to stimulate angiogenesis [10,115]. This temperature range is optimal for inducing HSP70-mediated cytoprotection and shifting macrophage polarization from M1 to M2 phenotypes without causing thermal damage [44,56].

Integration of Smart Surgical Materials implementing these protocols requires real-time monitoring at the tissue level, which is currently lacking in standard care. The development of “closed-loop” therapeutic systems represents the next frontier in abdominal surgery:

- Utilizing conductive hydrogels and nanowire-based sensors allows for continuous monitoring of wound temperature and microenvironment status [116]. Future iterations should incorporate immunosensors capable of detecting local inflammatory spikes. We envision a system where “smart” sutures not only detect local hypothermia but autonomously activate micro-heating elements to restore optimal enzymatic temperature. Preclinical studies suggest this technology can significantly reduce regeneration time and infection risks [117].

A key next step for the surgical community is to define the “therapeutic thermal window” for liver surgery specifically. Research should focus on quantifying the specific “thermal dose” required to maximize HSF1 activation without triggering NLRP3 inflammasome-mediated pyroptosis [101,110].

By integrating biomarker profiling with responsive biomaterials, surgeons could transition from passive temperature monitoring to active, immunologically optimized thermal wound management.

## 4. Materials and Methods

A comprehensive review of the literature was conducted on the molecular and immunological mechanisms of postoperative wound healing in the abdominal cavity, with particular focus on liver surgery. To this end, a systematic search of indexed scientific publications from 2010 to 2025 was performed in PubMed, Scopus, Web of Science, and Google Scholar, with attention to the reliability and relevance of the sources used.

To identify articles relevant to the study topic, combinations of keywords in both Polish and English were used, such as: “postoperative wound healing”, “abdominal surgical wounds”, “liver surgery wounds”, “thermal immunomodulation”, “heat shock proteins (HSP)”, “local tissue temperature”, “immune response modulation”, “thermal stress and inflammation”, “damage-associated molecular patterns (DAMPs)”, “wound healing and temperature”, “molecular mechanisms of tissue repair”, “thermotherapy in hepatic surgery”, and “HSP expression in wound healing”. English-language original and review articles were included, with particular emphasis on publications from the last decade. Inclusion criteria were: publications from the past 15 years (2010–2025), review articles and original studies on molecular mechanisms of wound healing after abdominal surgery, with a focus on liver procedures, studies addressing the role of temperature and heat shock proteins in tissue regeneration, research on postoperative wounds, particularly abdominal surgical wounds, studies discussing thermoimmunomodulation and the role of defensin peptides in wound healing. Exclusion criteria comprised publications limited to animal models without reference to human molecular processes and articles not available in full text.

### 4.1. Study Design

The present work is a narrative review summarizing current knowledge on thermal immunomodulation in postoperative wound healing, with a particular focus on hepatic surgery. The review synthesizes molecular, immunological, and clinical findings relevant to temperature-dependent modulation of wound repair processes.

### 4.2. Search Strategy

A structured literature search was performed in PubMed, Scopus, Web of Science, and Google Scholar, covering the years 2010–2025. The search strategy was based on combined Medical Subject Headings (MeSH) and free-text terms related to postoperative wound healing, local thermal regulation, and immune mechanisms. The following keywords and their combinations were used:

- “postoperative wound healing”, “abdominal surgical wounds”;
- “liver surgery wound repair”, “hepatic surgery”;
- “thermal immunomodulation”, “local tissue temperature”;
- “heat shock proteins”, “HSP70”, “HSP90”;
- “thermal stress and inflammation”;
- “DAMPs”, “danger-associated molecular patterns”;
- “TLR signaling”, “NLRP3 inflammasome”;
- “molecular mechanisms of tissue repair”;
- “thermotherapy in hepatic surgery”;
- “wound healing and temperature modulation”.

Manual searches of reference lists from key molecular-immunology and wound-healing articles were also performed to identify additional relevant publications.

### 4.3. Selection Process

The initial search yielded 312 publications. After removal of duplicates and screening of titles and abstracts, 118 papers were selected for full-text review. Following detailed evaluation, 82 studies were included in the final synthesis. The reviewed literature comprised:

- Original molecular and translational studies on temperature-dependent regulation of wound healing mechanisms (HSP activation, MAPK/NF-κB pathways, cytokine modulation);
- Clinical studies on postoperative hypothermia, normothermia, and local hyperthermia in abdominal and hepatic surgery;
- Review papers on wound immunology, DAMP signaling, inflammasome activation, and biomolecular repair mechanisms;
- Research addressing thermal stress responses, macrophage polarization, angiogenesis, and ECM remodeling.

#### 4.3.1. Inclusion Criteria

1. Publications from 2010 to 2025 in English or Polish.
2. Studies addressing:
  - Molecular and immunological pathways in postoperative wound healing;
  - Thermal regulation (hypothermia, normothermia, hyperthermia) and its impact on immune responses;
  - The role of heat shock proteins (HSP70, HSP90, HSP27) in tissue repair;
  - DAMP-related activation of TLR and inflammasome pathways;
  - Postoperative wounds after abdominal or liver surgery.
3. Original studies, narrative reviews, systematic reviews, meta-analyses, and clinically relevant experimental work.
4. Articles with clear relevance to human physiology or human-relevant mechanisms (e.g., translational studies on MAPK, NF-κB, TLR4, NLRP3 activation in wound healing).

#### 4.3.2. Exclusion Criteria

1. Studies performed exclusively on animal models without translational relevance to human tissue responses.
2. Publications lacking descriptions of molecular or immunological mechanisms (e.g., purely technical surgical reports).
3. Articles without accessible full texts.
4. Studies unrelated to postoperative wounds, liver surgery, or temperature-dependent immunomodulation.
5. Papers providing duplicated data or commentaries lacking scientific value.

### 4.4. Data Extraction and Synthesis

Extracted data included:

- Temperature ranges influencing wound-healing physiology;
- Mechanisms of HSP activation and cytoprotection;
- Cytokine profiles (TNF-α, IL-1β, IL-6);
- DAMP-related responses and inflammasome activation;
- Effects on angiogenesis, macrophage polarization, cellular proliferation, and ECM remodeling.

All findings were synthesized narratively, integrating molecular insights with clinical implications for postoperative hepatic wound management.

## 5. Conclusions

1. Local hyperthermia induces and modulates the expression of heat shock proteins (HSPs) and activates the NLRP3 inflammasome and Toll-like receptors (TLRs) via DAMP molecules, triggering a cascade of proinflammatory cytokines (IL-1β, IL-6, TNF-α) and the COX-2/PGE pathway. This raises the thermoregulatory set point, causing postoperative fever, while stabilizing immune cell functions and optimizing the balance between inflammation and tissue regeneration.
2. The pyrogenic response depends on the type and depth of the injury, allowing for tailored thermomodulation strategies in abdominal surgery.
3. Precise thermoimmunomodulation of abdominal surgical wounds enables individualized immune responses, supports effective healing, and minimizes the risk of postoperative complications.
4. Understanding these mechanisms provides a foundation for the development of innovative therapies aimed at treating both chronic wounds and postoperative wounds in the abdominal region.

## Figures and Tables

**Table 1:** Key factors and proteins involved in wound healing processes and modulation of immune response [50].

Abbreviation	Full Name	Biological Function
VEGF	Vascular Endothelial Growth Factor	It stimulates the formation of new blood vessels (angiogenesis).
PDGF	Platelet-Derived Growth Factor	It promotes the migration and proliferation of fibroblasts and smooth muscle cells.
TGF-β	Transforming Growth Factor Beta	It regulates the inflammatory response, cell differentiation, and extracellular matrix remodeling.
IL-6	Interleukin 6	A proinflammatory and regenerative cytokine; it activates T and B lymphocytes as well as hepatocytes.
TNF-α	Tumor Necrosis Factor Alpha	A key mediator of inflammation; it activates immune cells and induces apoptosis.
HSP	Heat Shock Proteins (HSP)	Protective chaperone proteins safeguarding against heat, chemical, and oxidative stress.
ECM	Extracellular Matrix (ECM)	A structural scaffold supporting cells; critical for cell migration, adhesion, and differentiation.
MMP	Matrix Metalloproteinases	Enzymes that degrade components of the extracellular matrix (ECM)—essential for tissue remodeling.
HSF	Heat Shock Factor	Regulates the expression of heat shock proteins (HSPs) in response to thermal stress.
TLR4	Toll-like Receptor 4	Participates in pathogen recognition and the initiation of the inflammatory response.
CD91	Cluster of Differentiation 91 (LRP1)	Receives signals from heat shock proteins (HSPs) and activates immune or protective responses.

**Table 2:** Body responses to increased temperature and the role of heat shock proteins (HSPs) according to the temperature range [52].

Temperature	Body Response	Nature of Response	Role of Heat Shock Proteins (HSPs)
~38 °C	Physiological stress	Adaptive	Mild activation, protein protection
39–40 °C	Fever, inflammation	Defensive	Strong activation, damage control
>40 °C	Hyperthermia	Pathological	Possible HSP insufficiency, cellular damage

**Table 3:** Heat shock proteins (HSP): classification, regulation, and functions in wound healing [72].

Class	Molecular Weight [kDa]	Function
HSP27	27	Actin stabilization, protection against oxidative stress
HSP70	70	Major heat stress response, inhibition of apoptosis
HSP90	90	Chaperone for receptor proteins (e.g., VEGF, TGF-β)
HSP110	110	Structural stabilization of extracellular matrix (ECM) proteins

**Table 4:** Activation of pyrogenic pathways following liver surgery [83,84].

Stage	Location/Event	Pathway/Molecule	Immunological Effect	Effect on Temperature
1	Surgical wound (liver tissue, skin)	Tissue damage → DAMPs (HMGB1, HSP, ATP)	Activation of antigen-presenting cells (macrophages, dendritic cells)	Initiation of inflammation
2	Hepatocytes, Kupffer cells, DCs	TLR4/TLR9	Release of proinflammatory cytokines: IL-1β, IL-6, TNF-α	Induction of fever via CNS action
3	Peripheral blood, liver	NLRP3 inflammasome	IL-1β maturation → strong activation of systemic febrile response	Temperature rise ~24–48 h post-surgery
4	Liver/plasma	IL-6 → IL-6R → STAT3	Stimulation of acute-phase proteins (CRP, fibrinogen), further support of fever	Maintenance of fever and immune system mobilization
5	Hypothalamus (central nervous system)	IL-1β/PGE2	Induction of COX-2 in CNS endothelial cells → elevation of set-point temperature	Thermoregulation change: systemic fever
6	Hepatic microcirculation, endothelium	TNF-α/IL-1β → endothelial activation → VCAM-1, ICAM-1	Recruitment of neutrophils, local tissue damage → further amplification of inflammatory cascade	Increase in local tissue temperature (microfever)
7	Liver/bone marrow	IL-6, G-CSF	Mobilization of monocytes and neutrophils → enhanced cellular response	Moderate fever persisting up to 72 h

**Table 5:** Course of transverse surgical access to the liver illustrated by a subcostal laparotomy [85,86].

Stage/Layer	Anatomical Structure	Type of Injury	Most Strongly Activated Molecules/Mechanisms
1. Dermis	Epidermis + dermis	Mechanical incision, superficial bleeding	HSP70, HMGB1, ATP, uric acid—activation of Langerhans cells and keratinocytes (TLR2, TLR4 receptors)
2. Subcutaneous tissue	Adipose tissue (panniculus adiposus)	Vessel cutting, adipocyte separation	Release of lipids, ATP, HSP60; local activation of tissue macrophages M1
3. Superficial fascia	Scarpa’s fascia (membranous layer of subcutaneous tissue)	Cutting of collagen and elastin fibers	Release of collagen-derived DAMPs (e.g., fibrin and elastin fragments)
4. Abdominal muscles	External oblique, internal oblique, transversus abdominis	Incision or detachment of muscle fibers	Myoglobin, ATP, HSPs, nuclear DNA—activation of monocytes and macrophages via TLR9, TLR4; NLRP3 inflammasome activation
5. Deep fascia	Transversalis fascia	Cutting of deep connective tissue	Release of ECM-DAMPs (laminin, fibronectin fragments), signaling to mast cells and neutrophils
6. Parietal peritoneum	Serous membrane lining the abdominal cavity	Incision and mesothelial layer injury	IL-1β, IL-6, rapid response of mesothelial and endothelial cells → PGE2 generation
7. Liver parenchyma (optional)	During resection: liver capsule (Glisson’s capsule), hepatocytes	Parenchymal incision, coagulation	HMGB1, mitochondrial DNA (mitDNA), ATP, Kupffer cell activation, strong expression of IL-6, TNF-α, ROS, NLRP3 inflammasome activation

## Data Availability

Data is contained within the article.

## Abbreviations

The following abbreviations are used in this manuscript:

VEGF	Vascular Endothelial Growth Factor
PDGF	Platelet-Derived Growth Factor
TGF-β	Transforming Growth Factor Beta
IL-6	Interleukin 6
TNF-α	Tumor Necrosis Factor Alpha
HSP	Heat Shock Proteins
ECM	Extracellular Matrix
MMP	Matrix Metalloproteinases
HSF	Heat Shock Factor
TLR4	Toll-like Receptor 4
CD91	Cluster of Differentiation 91
DAMPs	Damage-Associated Molecular Patterns
HMGB1	High Mobility Group Box 1
ATP	Adenosine Triphosphate
DC	Dendritic Cells
IL-1β	Interleukin-1 beta
NLRP3	NOD-Like Receptor Protein 3
STAT3	Signal Transducer and Activator of Transcription 3
CRP	C-Reactive Protein
PGE2	Prostaglandin E2
COX-2	Cyclooxygenase-2
VCAM-1	Vascular Cell Adhesion Molecule 1
ICAM-1	Intercellular Adhesion Molecule 1
G-CSF	Granulocyte Colony-Stimulating Factor
CNS	Central Nervous System
ECM-DAMPs	Extracellular Matrix–Derived Damage-Associated Molecular Patterns
ROS	Reactive Oxygen Species
mitDNA	Mitochondrial DNA
